# *Origanum syriacum* L. Attenuates the Malignant Phenotype of MDA-MB231 Breast Cancer Cells

**DOI:** 10.3389/fonc.2022.922196

**Published:** 2022-06-30

**Authors:** Amal AlKahlout, Manal Fardoun, Joelle Mesmar, Rola Abdallah, Adnan Badran, Suzanne A. Nasser, Serine Baydoun, Firas Kobeissy, Abdullah Shaito, Rabah Iratni, Khalid Muhammad, Elias Baydoun, Ali H. Eid

**Affiliations:** ^1^ Kurome Therapeutics, Cincinnati, OH, United States; ^2^ Department of Biology, American University of Beirut, Beirut, Lebanon; ^3^ Department of Basic Sciences, University of Petra, Amman, Jordan; ^4^ Department of Pharmacology and Therapeutics, Beirut Arab University, Beirut, Lebanon; ^5^ Department of Diagnostic Radiology, Cleveland Clinic, Cleveland, OH, United States; ^6^ Program for Neurotrauma, Neuroproteomics & Biomarkers Research, Departments of Emergency Medicine, Psychiatry, Neuroscience and Chemistry, University of Florida, Gainseville, FL, United States; ^7^ Biomedical Research Center, Qatar University, Doha, Qatar; ^8^ Department of Biology, United Arab Emirates University, Al Ain, United Arab Emirates; ^9^ Department of Basic Medical Sciences, College of Medicine, QU Health, Qatar University, Doha, Qatar

**Keywords:** *Origanum syriacum* L., ethnopharmaclogy, Za’atar, phytotherapy, breast cancer

## Abstract

Breast cancer is the leading cause of cancer-related deaths among women. Among breast cancer types, triple negative breast cancer (TNBC) is the most aggressive, and is resistant to hormonal and chemotherapeutic treatments. As such, alternative approaches that may provide some benefit in fighting this debilitating pathology are critically needed; hence the utilization of herbal medicine. *Origanum syriacum* L., one of the most regularly consumed plants in the Mediterranean region, exhibits antiproliferative effect on several cancer cell lines. However, whether this herb modulates the malignant phenotype of TNBC remains poorly investigated. Here, we show that in MDA-MB-231, a TNBC cell line, *Origanum syriacum* L. aqueous extract (OSE) inhibited cellular viability, induced autophagy determined by the accumulation of lipidized LC3 II, and triggered apoptosis. We also show that OSE significantly promoted homotypic cell-cell adhesion while it decreased cellular migration, adhesion to fibronectin, and invasion of MDA-MB-231 cells. This was supported by decreased activity of focal adhesion kinase (FAK), reduced α2 integrin expression, and downregulation of secreted PgE_2_, MMP2 and MMP-9, in OSE-treated cells. Finally, we also show that OSE significantly inhibited angiogenesis and downregulated the level of nitric oxide (NO) production. Our findings demonstrate the ability of OSE to attenuate the malignant phenotype of the MDA-MB-231 cells, thus presenting *Origanum syriacum* L. as a promising potential source for therapeutic compounds for TNBC.

## Introduction

Breast cancer remains the most frequent cancer among females, affecting 2.1 million women each year (1, 2). It is the leading cause of cancer-related mortalities accounting for approximately 15% of all cancer deaths among women (1–3). In addition, breast cancer rates are increasing in almost every region worldwide (1, 2).

Literature reported several classifications of breast cancer. Early classification depended on histological features (4). However, recent “microarray revolution” showed that breast cancer types differ in their mRNA expression profiles (5, 6). Consequently, breast cancer was categorized into molecular subtypes (4), with the triple-negative breast cancer (TNBC) being among the most aggressive types (7). Immunoistochemical analysis reveals that TNBC is characterized by the lack of expression of estrogen receptor (ER) and progesterone receptor (PR), in addition to an absence of human epidermal growth factor receptor 2 (HER2) protein overexpression (8). These three receptors are the most commonly targeted biomarkers, and their absence renders most conventional hormonal and chemotherapeutic treatments relatively ineffective (9). This is reflected by the poor prognosis and low 5-year survival rate of TNBC patients (10). In addition, most TNBC cells evade apoptosis and acquire chemoresistance as well as avoid DNA repair mechanisms (11). It is well known that chemotherapy is often associated with many undesirable and severe side effects (12). Taken together, these observations mandate that alternative approaches, such as herbal medicine, be sought to develop novel treatments that could be more efficacious or exerts less undesired effects. Indeed, recent studies focus on herbal medicine as a treatment of several diseases, including aggressive breast cancer (13–22). In addition, a large percentage of women appear to be highly interested in using herbal remedies to prevent or treat breast cancer (23, 24).

*Origanum syriacum* L. is an aromatic perennial herb belonging to the mint family, Lamiaceae (25). It is native to the Mediterranean region, especially Lebanon, where it is used in culinary and other preparations (26). Its various medical effects were long described in Avicenna’s “The Canon of Medicine” (980 – 1037 AD) (26). These effects are likely due to the richness of *Origanum syriacum* L. in bioactive compounds such as terpinene, carvacrol, p-cymene, thymoquinone, thymol, and β-caryophyllene (25, 26). These compounds were reported to have anti-inflammatory (27), antimicrobial (28), anti-glycemic (29), and anticancer (30) effects. In addition, many of these bioactive compounds exhibit antioxidant effects (31) known to inhibit the malignant phenotype of human breast cancer cells (32, 33). Indeed, *Origanum syriacum* L. showed strong anti-proliferative activities against THP-1 human leukemia cells (34), LoVo, SW620, HCT 116, and Vero colon cancer cells (35, 36), and MCF-7 breast cancer cells (37). Furthermore, the anticancer effect of other *Origanum* species such as *Origanum acutidens, Origanum vulgare, and Origanum majorana* on MDA-MB-231, a highly aggressive and metastatic breast cancer cells, was also reported (38–40). However, the anticancer effects of *Origanum syriacum* L. on MDA-MB-231, which represents an excellent representative model of TNBC, are still poorly investigated.

In this study, we evaluated the effect of *Origanum syriacum* L. aqueous extract on the malignant phenotype of a battery of breast cancer cell lines, but we focus on MDA-MB-231 cells as a classic representative of TNBC. Our results showed that OSE potently inhibited the proliferation, migration, invasion and adhesion of MDA-MB-231. In addition, OSE induced autophagy and apoptosis, and showed an anti-angiogenic effect. These results highlight the promising therapeutic potential of *Origanum syriacum* L. against invasive TNBC.

## Materials and Methods

### Cell Culture and Reagents

Cell lines were purchased from American Type Culture Collection (ATCC number: HTB-26™)(Manassas, VA, USA). Cells were cultured in DMEM media supplemented with 10% FBS, 1% penicillin/streptomycin and 2mM L-Glutamine. Cells were maintained in a humidified incubator at 37°C with 5% CO_2_ atmosphere. LC3I/II antibody, HRP-conjugated Goat Anti-Mouse antibody, HRP-conjugated Goat Anti-Rabbit antibody were purchased from Abcam (Cambridge, UK). Anti-GAPDH antibody, anti-caspase-3, were obtained from Cell Signaling Technology (Leiden, The Netherlands). Methylthiazolyldiphenyltetrazolium bromide (MTT) was purchased from Sigma-Aldrich (Schnelldorf, Germany). PgE_2_ and NO colorimetric ELISA quantification kits were purchased from Cayman Chemical (Ann Arbor, Michigan, USA). MMP-2 and MMP-9 ELISA quantification kits were obtained from R&D Systems (Minneapolis, MN, USA).

### Preparation of *Origanum syriacum* Aqueous Extract (OSE)

*Origanum syriacum* L. leaves were obtained from local farm (South of Lebanon) in the Spring season. The plant identity was confirmed by a plant taxonomist at Lebanese International University, Beirut, Lebanon, and the nomenclature is checked with http://www.theplantlist.org. Dried plant and its leaves were donated to the herbarium located at Qatar University. The *Origanum syriacum* aqueous extract was prepared as previously described (36). Briefly, leaves were rinsed to remove dust particles and then air-dried in a dark room. Then, leaves were ground into a fine powder using a porcelain mortar and pestle. The powder was suspended in distilled water (1:3 weight to volume ratio) and stirred overnight with continuous shaking. The mixture was then was vacuum-filtered and dried using a rotary evaporator. The extract was then lyophilized and kept at -20°C for further use. For cell treatment, OSE was diluted in culture media and sterilized using 0.2 µm filter.

### MTT Assay

Cells were seeded in 96-well plate at a concentration of 10^4^ cells/well. The following day, cells were treated with increasing concentrations of OSE (0.8, 1.2 and 1.6 mg/ml) for 24, 48, and 72 hours. MTT solution (20 µL, 5 mg/mL) was added to each well, and cells were incubated for an hour in a 5% CO_2_ incubator. Then the medium was removed and 200 µL of 100% DMSO was added into each well. The plates were then shaken for 15 min before reading the absorbance at 570 nm using TECAN M200 Pro (Tecan Group Ltd., Austria) microplate reader. Absorbance is directly proportional to cell viability.

### Wound Healing Assay

Cells were grown in 12-well plates until 90–95% confluent. The monolayer was then scratched using a yellow (20–200 µL) tip. Wells were washed three times with phosphate buffered saline (PBS) to remove cellular debris, then medium with or without OSE (1.2 mg/mL) was added into the wells. Wound healing was monitored, and photomicrographs were taken using Olympus IX53 inverted microscope (OLYMPUS Co., Tokyo, Japan). The width of the scratch was measured using Olympus cell Sens software (OLYMPUS Co, Tokyo, Japan).

### Transwell Migration Assay

Cells (5 x 10^4^/well) were seeded in the upper chamber of an insert (BD Biosciences, USA) in serum-free media, with or without OSE (1.2 mg/mL). This concentration was chosen since the time needed for this experiment must be short enough so as not to induce cell toxicity. As such, using the lower concentration of 0.8 mg/ml, even if effective, would complicate the results as it would need more than 24 hours, by which time, decreased viability may interfere with the results. The lower chamber was loaded with complete medium (containing 10% FBS), with this FBS acting as a chemoattractant. Cells were allowed to migrate for 6 hours; then they were washed with PBS and fixed with formaldehyde. After washing, migrated cells were stained with 1% crystal violet for 10 minutes, then were washed twice with PBS. Cells from at least five different random fields were counted under an inverted microscope (Objective x10). The number of migrated cells is presented as means ± SEM.

### Matrigel Invasion Assay

Transwell inserts were coated with matrigel and allowed to dry overnight. Cells in serum-free media were seeded onto the rehydrated upper transwell chamber in the absence or presence of OSE (1.2 mg/mL). The lower chamber was loaded with complete medium (serum acted as a chemotactic attractant). Following incubation for 24 hours, the medium was aspired and cells on the upper side of the insert membrane were removed with a cotton swab. Invading cells on the lower side of the membrane were fixed with methanol and stained with DAPI. The membrane was then cut with a blade and mounted on an anti-fade agent. Cells from at least five different random fields were counted.

### Cell Adhesion to Fibronectin

Wells of 96-well plates were coated with fibronectin and incubated at 37°C overnight. The following day, wells were blocked with 3% Bovine Serum Albumin (BSA) for 3 hours at room temperature. Cells (5× 10^3^/well) were then seeded and incubated for 60 minutes in the humidified incubator (37°C with 5% CO_2_). Nonadherent cells were removed by washing with PBS (3x) and adherent cells were stained with crystal violet and observed under the microscope. At least five random fields were counted and the experiment repeated three times.

### Quantification of Secreted of MMP-2, MMP-9 and PgE_2_

MDA-MB-231 cells (2× 10^5^/well) grown to a sub-confluent level in 6-well plate, then cultured for 24 hours in the presence or absence of OSE. The conditioned medium from OSE (0.8 and 1.2 mg/mL) treated cells and untreated cells was collected and the levels of secreted MMP-2, MMP-9 (Invitrogen, Camarillo, CA, USA) or PgE_2_ (Cayman Chemical, MI, USA) were determined using colorimetric ELISA kitsas per the manufacturer’s instruction. The optical density of each sample was measured using an AMP Platos R 496 microplate reader (AMP Diagnostics, Poland). The proteins present in the conditioned media were concentrated using the Amicon Ultra-0.5 protein purification and concentration column (Millipore) and protein concentration was assayed using the BCA protein assay kit (Thermo Scientific). Levels of the PgE_2_ and MMPs were normalized to the total protein level in each sample.

### Quantification of Nitrate/Nitrite Production

The amount of Nitrate/Nitrite production was determined with a colorimetric ELISA kit (Cayman Chemical, Ann Arbor, Michigan, USA), which is based on the Griess reaction, according to the manufacturer’s instructions. Briefly, this kit utilizes the conversion of nitrate to nitrite, followed by the conversion of this nitrite to a deep purple azo product that can be measured photometrically. The value of nitrate/nitrite presented is the total value measured in the well treated without or with OSE (0.8 and 1.2 mg/mL) for 24h, minus the value determined from the media alone in the absence of any growing cells. Assays were performed in triplicates and three independent experiments were performed.

### Chorioallontoic Membrane (CAM) Assay

Fifteen fertilized eggs were incubated while rotating at 37°C with 50% humidity for 6 days (E6). Then, a small window was cut in the eggshell above the CAM. The window was loaded with or without OSE, sealed with parafilm, and incubated at 37°C with 50% humidity. Images were taken at 0 hours and after 24 hours after treatment. Vessels area and number of junctions were quantified using AngioTool software.

### Western Blotting

Cells were washed with PBS and then lysed using lysis buffer (2% SDS, 60 mM Tris, pH 6.8) as we previously described (41). Following protein quantification, equal amounts of protein (20–30 µg) were resolved onto 5–11% SDS-PAGE. Proteins were then transferred onto polyvinylidene difluoride (PVDF) membrane (Biorad). The membrane was then blocked (5% fat-free milk in TBS-T, 1 hour at room temperature) and incubated overnight with the relevant primary antibody at 4°C. The membrane was then washed with TBS-T (3x 10 minutes) and incubated with the appropriate HRP-conjugated secondary antibody (1 hour, room temperature). The membrane was washed again with TBS-T (3x 10 minutes) and immunoreactive bands were detected by ECL chemiluminescent substrate (ECL clarity, Biorad) and quantified using Chemidoc MP Imaging system (Biorad).

### Caspase 3/7 Activity Assay

As we previously reported (42), cells (5,000/well) were seeded in triplicates in 96-well plates and treated without or with OSE. Caspase 3/7 activity was measured using a Caspase 3/7 Glo^®^ kit (Promega Corporation, USA) as per the manual’s instructions. Luminescence was measured, and data plotted as mean ± SEM for three independent experiments.

### Statistical Analysis

Results were expressed as means ± S.E.M. of the number of experiments. A Student’s *t*-test for paired or unpaired values was performed and a *p* value of <0.05 was considered statistically significant.

## Results

### OSE Inhibits the Proliferation of a Battery of Breast Cancer Cells

**Figure 1:**
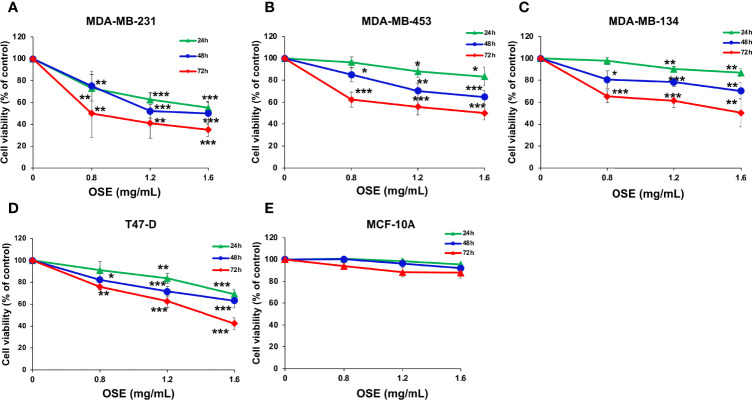
OSE Inhibits proliferation of a battery of breast cancer cell lines (MDA-MB 231 **(A)**, MDAMB-453 **(B)**, MDAMB-134 **(C)** T47-D **(D)**, without significantly inducing cytotoxicity in normal human mammary epithelial cells (MCF10A) **(E)**. Cells were treated with increasing concentrations of OSE (0.8, 1.2 and 1.6 mg/ml) for 24, 48, and 72 h Cell viability was assessed by the overall metabolic activity measured by MTT. Values are calculated as % of the corresponding vehicle control value and represented as mean ± standard error of the mean (SEM) of three independent experiments, each run in triplicate. (*p < 0.05, **p < 0.01 and ***p < 0.005).

We first examined the effect of *Origanum syriacum* aqueous extract (OSE) on cellular viability of MDA-MB-231 cells. Cells were exposed to increasing concentrations of OSE for 24, 48 and 72 hours and cell viability was examined as described in materials and methods. We found that OSE significantly reduced the viability of these cells in concentration- and time-dependent manner (**Figure 1A**). The IC_50_ is 1.69, 1.47 or 0.8 mg/mL at 24, 48 or 72 hours, respectively. Similar results were obtained in other breast cancer cell lines including T-47D (ER^+^, PR^+/-^, HER2^-^), MDA-MB-453 (ER^-^, PR^-^, HER2^+^), MDAMB-134 (ER^+^, PR^-^, HER2^-^) (**Figure 1**). Importantly, OSE did not significantly affect viability of the normal human mammary epithelial cell line, MCF-10A, which was used as a control cell line (**Figure 1**)

### OSE Induces Autophagy and Apoptosis in MDA-MB-231 Cells

**Figure 2:**
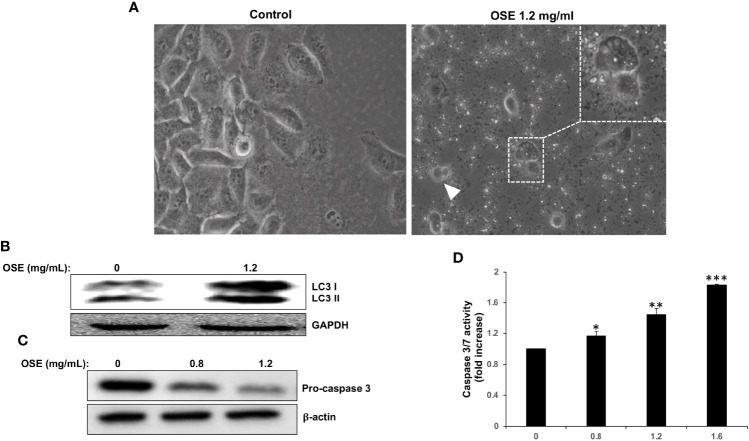
OSE induces autophagy and apoptosis in MDA-MB-231. **(A)** Cells were treated with OSE (1.2 mg/ml) for 48 hrs. Micrographs were captured at magnifications of 200X. **(B)** Induction of autophagy by OSE. Cells were treated with OSE (1.2 mg/ml) for 48 hrs, and cytoplasmic (LC3 I) and lipidized LC3 II accumulation was detected by Western blotting. **(C)** Activation of apoptosis by OSE. Cells were treated with OSE (0.8 and 1.2 mg/ml) for 48 hrs, and cleavage of procaspase-3 was examined by Western blotting. **(D)** Cells were treated without (0) or with OSE (0.8, 1.2 or 1.6 mg/ml) for 24 hours, and then Caspase 3/7 activity measured. (* denotes p<0.05, ** denotes p<0.01, and *** denotes p<0.001).

Light microscopy observation of MDA-MB-231 cells treated with OSE revealed cytoplasmic vacuolation (**Figure 2A**, thin arrow) in subpopulation of treated cells, suggestive of autophagy in those cells. In addition, some treated cells appeared smaller and rounded, characteristic of dying cells (**Figure 2**, arrow head). In order to determine whether the cytoplasmic vacuolation resulted from activation of autophagy, we examined by Western blotting the accumulation of the lipidized LC3 II a marker of autophagosome formation. As shown in **Figure 2B**, OSE induced the accumulation of LC3 II, hence arguing in favor of autophagy induction in treated cells. Next, we looked whether apoptosis is also activated in response to OSE. We found that OSE induces a concentration-dependent cleavage of the effector procaspase 3 in MDA-MB-231 cells (**Figure 2C**). In addition, OSE, in a concentration and time-dependent manner, significantly increased the activity of Caspase 3/7, a major apoptosis-executing enzyme (**Figure 2D**). Altogether, our date suggests that OSE inhibits breast cancer through induction of autophagy and activation of apoptosis.

### OSE Attenuates the Migration and Downregulates Activated Focal Adhesion Kinase in MDA-MB-231 Cells

**Figure 3:**
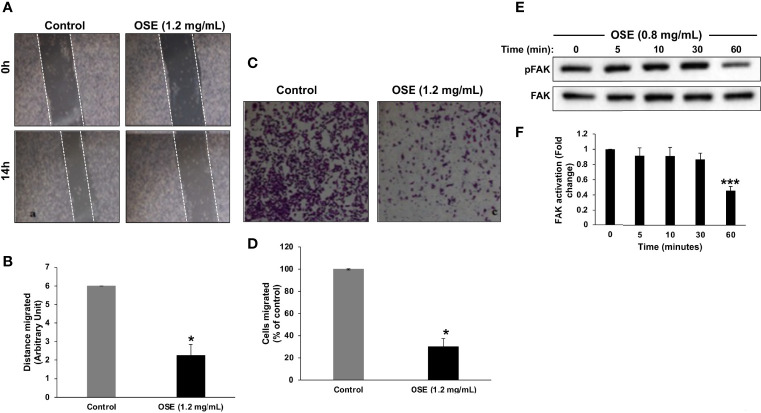
OSE inhibits migration and downregulates activated FAK in MDA-MB-231 cells. **(A, B)** Cells were treated with OSE (1.2 mg/ml) and cell migration was assessed by wound healing assay. **(C, D)** Inhibition of cell migration in transwell migration assay. Images of crystal violet-stained migratory cells were taken 6 h post-treatment. Values are represented as mean ± SEM of distance migrated (n = 3 replicates) (*p < 0.05). **(E)** Downregulation of pFAK by OSE. Cells were treated with OSE (0.8 mg/ml) for 5, 10, 30 and 60 mins, and the phosphorylation level of FAK was determined by Western blotting. **(F)** Quantitation of three independent replicates plotted as mean of fold change (***p < 0.001).

Next, we examined the effect of OSE on the migratory potential of cells. Results of wound healing assay showed that OSE significantly attenuated the migration potential of MDA-MB-231 cells (**Figures 3A, B**). OSE-mediated inhibition of migration was further confirmed by transwell migration assay. As shown in **Figures 3C, D**, the number of OSE-treated cells was dramatically reduced by ~60% compared to control cells. It is important to mention here that the timepoint chosen for this assay (i.e. 6 hours post-treatment) was so that we can be sure that any decrease in migration is not due to decrease in viability. Indeed, at this timepoint, there was no decrease in viability and no increase in caspase activity (data not shown).

It is well documented that the activation of focal adhesion kinase (FAK) is associated with cell adhesion and migration of various cancer cell lines. Thus, we sought to determine the effect of OSE on FAK activity. Western blotting analysis revealed that a low concentration of OSE (0.8 mg/mL) significantly downregulated the level of activated FAK (pFAK) as early as 1 hour post-treatment (**Figures 3E, F**). Our data demonstrate that OSE inhibits the migration potential of MDA-MB-231 cells most likely through inactivation of FAK.

### OSE Inhibits Adhesion to Fibronectin and Downregulates Integrin- α2 in MDA-MB-231 Cells

**Figure 4:**
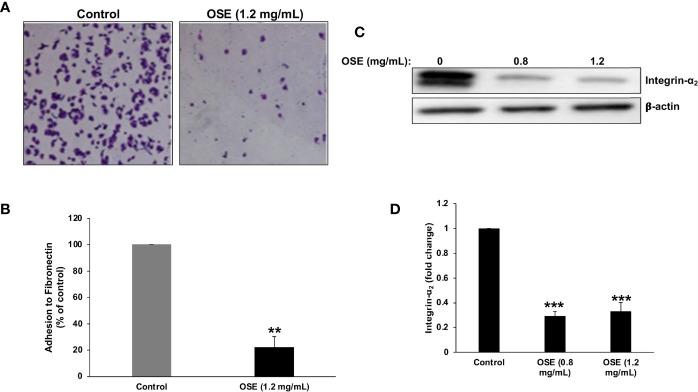
OSE inhibits adhesion of MDA-MB-231 cells to fibronectin and downregulates integrin α2. **(A, B)** OSE impairs the ability of MDA-MB-231 cells to adhere to fibronectin. Cells were treated with OSE (1.2 mg/ml) and allowed to adhere to fibronectin for 1 hr. Representative photomicrographs show the effect of OSE on MDA-MB-231 adhesion. Values are represented as mean ± SEM of relative fold inhibition of control cells (**p < 0.005). **(C, D)** Cells were treated with OSE (0.8 mg/ml) for 24h, and the level of integrin α 2 was detected by Western blotting. Values represent mean fold change of three independent replicates. One-way ANOVA was performed (***p < 0.001).

Cellular adhesion to the ECM is a crucial step for migration and invasion of cancer cells. So, we examined the effect of OSE on the ability of MDA-MB-231 to adhere to fibronectin, a key component in the extra cellular matrix (ECM). As it is shown in **Figures 4A, B**, OSE significantly inhibited the adhesion of MDA-MB-231cells to fibronectin by ~78%. This is supported by our finding that untreated cells exhibit significantly reduced adhesion to plates coated with RGD, a peptide known to mediate cell adhesion to fibronectin (data not shown).

Integrins, including integrin α2, are major mediators of cell adhesion of invasive breast cancer to fibronectin. Having shown that OSE inhibits the adhesion of MDA-MB-231 cells to fibronectin, we sought to determine whether this inhibition involves disruption of the integrin-fibronectin interaction. Toward this, we measured, by western blotting, the effect of OSE on the protein level of integrin α2. As shown in **Figures 4C, D**, OSE significantly reduced the level of integrin α2 in OSE-treated cells and therefore strongly suggest that the anti-adhesive effect of OSE is potentially mediated by downregulation of integrin α2 in MDA-MB-231 cells.

### OSE Induces Aggregation of MDA-MB-231 Cells

**Figure 5:**
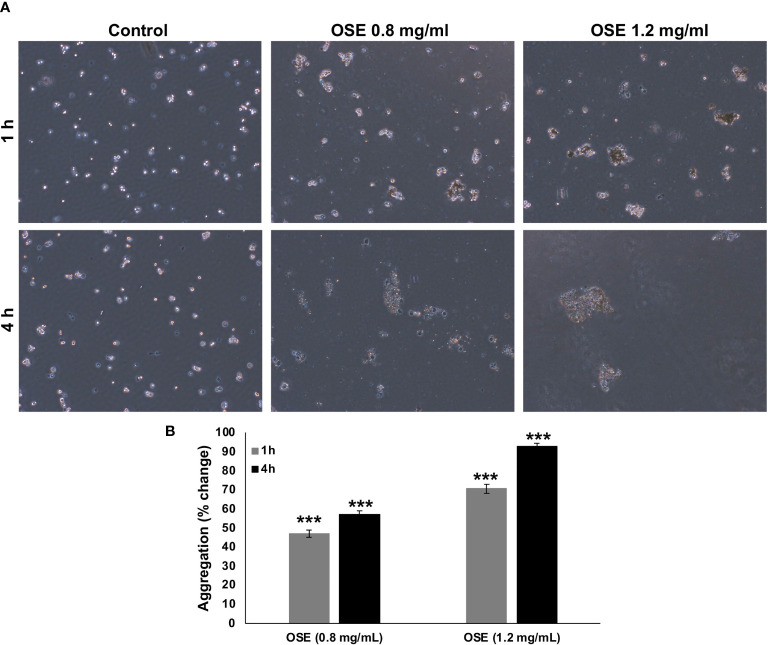
OSE promotes MDA-MB-231 cell-cell aggregation. **(A, B)** Cells were incubated with or without OSE treatment (0.8 and 1.2 mg/ml) for 1 or 4h and subjected to aggregation assay. Percentage aggregation was calculated according to the following formula: % aggregation = (1-Nt/Nc) x 100, where Nt and Nc represent the number of single cells in OSE-treated or control groups respectively. Values are represented as mean ± standard error of the mean (SEM) of three independent experiments (***p < 0.001).

Epithelial-mesenchymal transition (EMT) has been implicated in conferring metastatic properties to cancer cells. Hallmarks of EMT includes loss of cell connection between neighboring cells. To examine whether OSE cell-cell homotypic adhesion, aggregation assay was carried out. OSE treatment was able to promote aggregation in a time- and concentration- dependent manner of MDA-MB-231 cells (**Figure 5**).

### OSE Inhibits Invasion of MDA-MB-231 Cells and Downregulates the Level of Secreted MMP-2, -9 and PgE_2_

**Figure 6:**
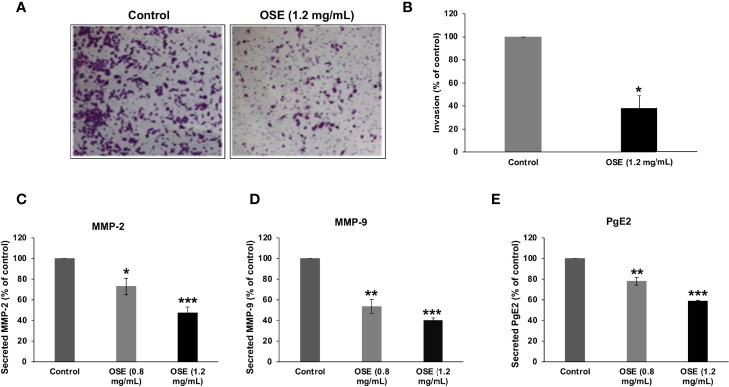
OSE inhibits invasion and downregulates MMP-2, MMP-9 and PgE2 in MDA-MB-231 cells. **(A, B)** Cells were treated with OSE (1.2 mg/ml) and cell invasion was evaluated using invasion assay as described in materials and methods. Representative photomicrographs show the effect of OSE on invading cells. (**C–E**) Cells were grown in with (0.8 and 1.2 mg/mL) or without OSE. Levels of secreted MMP-2 **(C)**, MMP-9 **(D)**, and PgE2 **(E)** were evaluated by ELISA as described in material and methods. Values are represented as mean ± standard error of the mean (SEM) of three independent experiments (*p < 0.05, **p < 0.005, ***p < 0.001).

Next, we evaluated the effect of OSE on the invasive capacity of MDA-MB-231 cells in Matrigel-coated Boyden chambers. Our results show that OSE inhibited cell invasion, evident by the significant decrease by 60% in number of OSE-treated cells that has passed through the Matrigel coated membrane (**Figures 6A, B**).

Degradation of the ECM by matrix metalloproteinases (MMP) is an important event in cancer invasion and metastasis. Thus, we sought to evaluate the effect of OSE on MMP-2 and MMP-9 activity. We found that OSE decreased, in concentration-dependent manner, the levels of secreted MMP-2 (**Figure 6C**) and MMP-9 (**Figure 6D**) in MDA-MB-231 cells.

Prostaglandin E2 (PgE_2_), highly expressed in breast tumors, was reported to regulate cell growth, migration and invasion of several tumors. We found that OSE was also able to significantly reduce the level of PgE_2_ in MDA-MB-231 cells (**Figure 6E**). Altogether, our findings strongly suggest that downregulation of MMP-2, MMP-9 and PgE_2_ secretion account, although may be not solely, to the impaired invasive ability of OSE-treated MDA-MB-231 cells.

### OSE Inhibits *In Ovo* Angiogenesis

**Figure 7:**
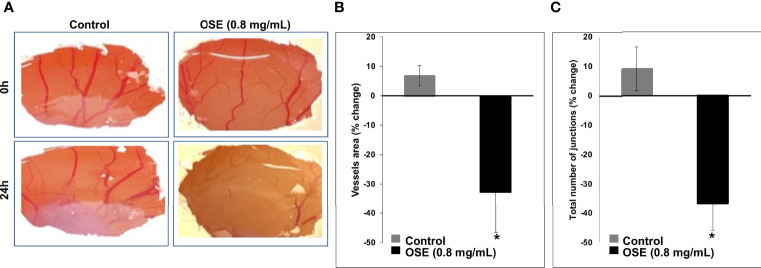
OSE inhibits angiogenesis *in ovo*. OSE (0.8 mg/ml) was added on the CAM of E6 embryo. Photographs **(A)** taken 24h after treatment show the change in vessels area **(B)** and the total number of junctions **(C)**. Values are represented as mean ± SEM (*p < 0.05).

Angiogenesis is crucial for tumor development and metastasis. Thus, we sought to determine whether OSE could affect the formation of new blood vessels using chick chorioallantoic membrane (CAM) angiogenesis assay, a powerful tool for this objective. **Figure 7A** shows that OSE (0.8 mg/mL) significantly decreased not only the vessels area but also the number of junctions in OSE-treated compared to control embryos. Indeed, the vessel area dropped from 6.85 ± 3.49% in control to -32.68 ± 13.75% in OSE-treated embryo (**Figure 7B**). The number of junctions were also reduced from 9.12 ± 7.45% in control to -36.68 ± 9.14% in OSE-treated embryo (**Figure 7C**). These results demonstrated that one possible mechanism through which OSE might exert its anti-breast cancer activity is through inhibition of angiogenesis.

### OSE Reduces Nitric Oxide Production in OSE-Treated MDA-MB-231 Cells

**Figure 8:**
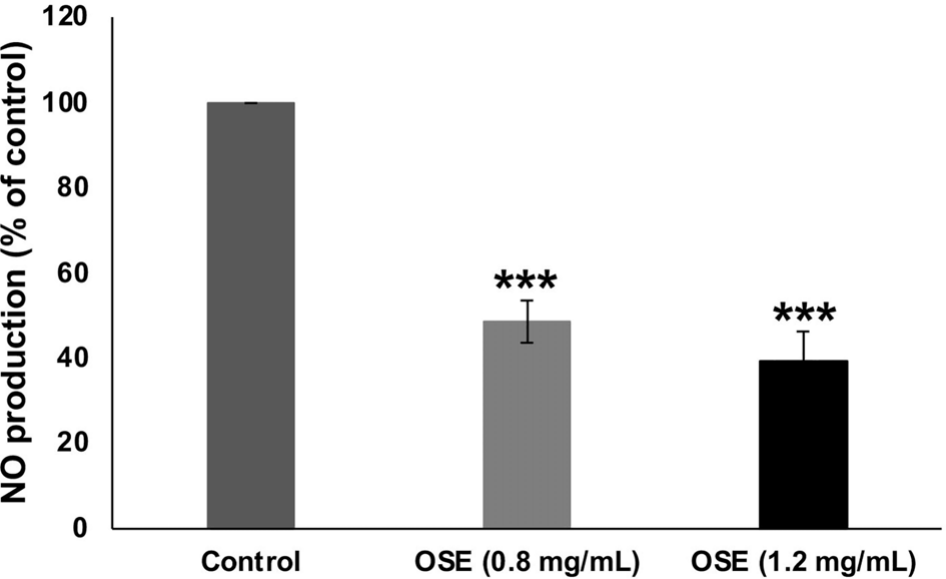
OSE reduces NO in MDA-MB-231 cells. MDA-MB-231 cells were treated with OSE (0.8 and 1.2 mg/ml) for 24h and Nitrate/Nitrite production was quantified as described in Materials and Methods. Values are represented as mean ± SEM (***p < 0.001).

Nitric oxide (NO) signaling was shown to promote breast tumor progression and metastasis by altering the expression of genes implicated in cellular migration, invasion and angiogenesis. Having shown that OSE inhibited migration, invasion and angiogenesis in MDA-MB-231 cells, we decided then to examine the effect of OSE on the NO production in those breast cancer cells. As shown in **Figure 8**, OSE significantly decreased NO production in a concentration-dependent manner in MDA-MB-231 cells. Based on this finding, we can hypothesize that OSE could also exert its anti-metastatic effect on breast cancer cells though modulation of NO production.

## Discussion

Breast cancer is the most common incident cancer (43), with the TNBC accounting for 10% to 15% of all breast cancer types (44). TNBC is irresponsive to hormonal therapies and acquires resistance to chemotherapeutic agents, making TNBC treatment a real challenge. A potential candidate for TBNC is herbal medicine, especially that many prescribed anticancer drugs are of plant origin (45). In this study we assessed the effect of *Origanum syriacum* on the malignant phenotype of MDA-MB-231, a TNBC cell line. Our results showed that OSE inhibited the proliferative, migratory, invasive and adhesive capacities of MDA-MB-231 cells. In addition, it efficiently inhibited angiogenesis *in ovo*. Hence, OSE targets several key contributors to breast cancer malignancy, validating not only its potential medicinal use against TNBC but also making it a potential source for identification of new phytochemicals with potent anticancer activity.

The significance of this study arises from the relevance of the used model and the availability of the used plant. Knowing that TNBC has higher proliferative, migratory and invasive characteristics; it was of obvious importance and more clinical relevance to use the MDA-MB-231 as our model. Another important point is the availability of *Origanum syriacum* in a region with high incidence of breast cancer. Indeed, this plant native is to some Arab countries, where the average onset of breast cancer in Arab women is 10 years younger than in American and European women (46). As such, this study opens a new horizon to accessible cost-effective breast cancer therapy in a high incident region.

The major hallmark of cancer is uncontrolled cell proliferation, which is markedly exacerbated in TNBC. Our results showed that OSE reduced proliferation of MDA-MB-231 cells. Other plant species belonging to the *Origanum* genus were also reported to have cytotoxic effect on MDA-MB-231 cells. These include *Origanum acutidens, Origanum vulgare, and Origanum majorana* (38–40). Apoptosis is a crucial process that aims at getting rid of abnormal cells (47). Malignant cells, including TNBC cells, can evade apoptosis and acquire chemoresistance (48). Thus, inducing apoptosis is an efficient approach in cancer treatment. When tumor cells escape apoptosis, autophagy appears as an alternative mechanism for cell death (49). Autophagy attenuates malignancy by promoting cancer cell death (50). Our results show that the antiproliferative effect of OSE was due to the induction of both, autophagy and apoptosis. This was concomitant with the downregulation of procaspase-3 and accumulation of lipidized LC3I/IIproteins. Although autophagy may be an escape or resistance mechanism for some cancers, it is not likely to be the case in these OSE-treated MDA-MB-231 cells. This is due to the notion that the other hallmarks of malignancy tested here (viability, migration, adhesion, aggregation, angiogenesis) are suppressed by OSE, likely indicating that these cells are executing a death pathway, rather than acquiring resistance.

Our results are in accordance with a several studies showing that the cytotoxic effect of anticancer herbs is mediated by autophagy and apoptosis (19). For instance, carnosol, a herbal compound, elicited its cytotoxic effects on MDA-MB-231 cells by inducing autophagy and apoptosis (51). Two *Origanum* species, *Origanum acutidens* and *Origanum majorana*, showed antiproliferative effects in MDA-MB-231 by inducing cell apoptosis (39, 42). In fact, *Origanum majorana* induced autophagy and apoptosis of colorectal cancer cells (HT-29 and Caco-2) (52, 53). This effect was mediated by p38 activation (53). Whether this same activation takes place to induce apoptosis in MDA-MB-231 cells is still to be determined. On the other hand, *Rhus coriaria* and tetrandrine, a compound extracted from *Stephania tetrandra*, induced autophagy in MDA-MB-231 (49, 54). This autophagy was achieved *via* inhibiting the PI3K/AKT/mTOR pathway by tetrandrine (49) and by inhibiting NFκB, STAT3 and nitric oxide pathways by *Rhus coriaria* (54). Whether *Origanum syriacum* promotes MDA-MB-231 autophagy by inhibiting these signaling pathways is yet to be investigated.

Patients with metastatic TNBC have a poor prognosis (55). This is related to the strong invasive and migratory abilities of TNBC cells (56). These events require ECM degradation by proteases, including MMPs. In fact, MMP upregulation results in reinforced migration, whereas their downregulation weakens migration (57). Interestingly, the expression of MMP-2 and MMP-9 is upregulated in breast cancer, and their expression level is correlated with lymph node metastasis and tumor staging (58). In our study, OSE attenuated the migration and invasion of MDA-MB-231 cells. This was accompanied by inhibition of FAK and downregulation of MMP-2 and MMP-9. These events likely underlie the compromised migratory and invasive capabilities. In addition, OSE inhibited cell adhesion. Thus, by hindering cell adhesion, migration, and invasion, OSE may potently inhibit cancer metastasis. In accordance, we have previously shown that another species of Origanum, namely *Origanum majorana*, inhibits migration and invasion of the MDA-MB-231 cells and decreases their adhesion to endothelial cells (59). These effects were accompanied by suppressing the activities and expression of MMP-2 and MMP-9 (59). Other plants such as *Rhus coriaria* also attenuated the migration, invasion, and blocked adhesion to fibronectin of MDA-MB-231 cells likely by downregulating MMP-9 (54).

PgE_2_ production, upregulated in breast cancer, is a marker for its progression (60). In fact, PgE_2_ is involved in almost all cancer processes including tumor development, migration, invasion, angiogenesis, and immunosuppression (61–63). Our results showed that OSE reduced secretion of PgE_2_. This downregulation may contribute to disseminating the previously described inhibitory effects of OSE on cell proliferation, migration, and invasion. Our results are in line with the *Rhus coriaria*-induced downregulation of PgE_2_, which likely underlies the anti-migratory, anti- invasive and anti-adhesive effects of the plant (54). It is worth mentioning that reducing PgE_2_ secretion by inhibiting COX-2 resulted in greatly compromised migration and invasion of MDA-MB-231 cells (64). As such, inhibitors of COX-2/PgE_2_ may be present in *Origanum syriacum* and may be used to suppress tumorigenicity of breast cancer.

The association of TNBC with epithelial–mesenchymal transition (EMT) is well established (65). During EMT, cancer cells gain migratory advantage *via* the disruption of cell-to-cell adhesion (66). Indeed, cells with reduced cell-cell adhesive capacity tend to be more migratory, invasive and malignant. Interestingly, OSE reversed the EMT phenotype by promoting cell aggregation as well as attenuating cell migration and adhesion to ECM, particularly the RGD domain of fibronectin. Knowing that drug resistance is associated with the expression of an EMT phenotype (67), it is not unreasonable to speculate that OSE potently reverses chemoresistance.

Angiogenesis promotes tumor vascularization and is a marker of tumor development and metastasis (68, 69). Invasive human breast cancers express several angiogenic factors, with vascular endothelial growth factor (VEGF) being the predominating factor (70). Patients with TNBC upregulated levels of VEGF and poor prognosis (71). OSE inhibited angiogenesis evident by decreased vessel length and junction number. This inhibition is probably mediated by the downregulation of the major angiogenic factor, VEGF. We have previously shown similar inhibitory effect with *Origanum majorana* (59). Furthermore, *Rhus coriaria* inhibited angiogenesis by reducing the production of VEGF in MDA-MB-231 (54). This anti-angiogenic potential of OSE is further cemented by our observation that it also inhibits NO, a gasotransmitter with pro-angiogenic capacity. It is important to mention here that we opted to use the 0.8 mg/ml rather than the 1.2 mg/ml due to the potential toxicity of the extract on the very sensitive developing embryo. Indeed, at a 0.8 mg/ml, it is obvious that a dramatic decrease in vascularization was noted. Thus, it would only be expected that a higher dose would so severely hamper vasculogenesis and angiogenesis that the sensitive embryo may die, rendering any finding at such a high dose rather obsolete. Together, this anti-angiogenic ability of *Origanum syriacum* further supports its potential as an anticancer drug.

**Figure 9:**
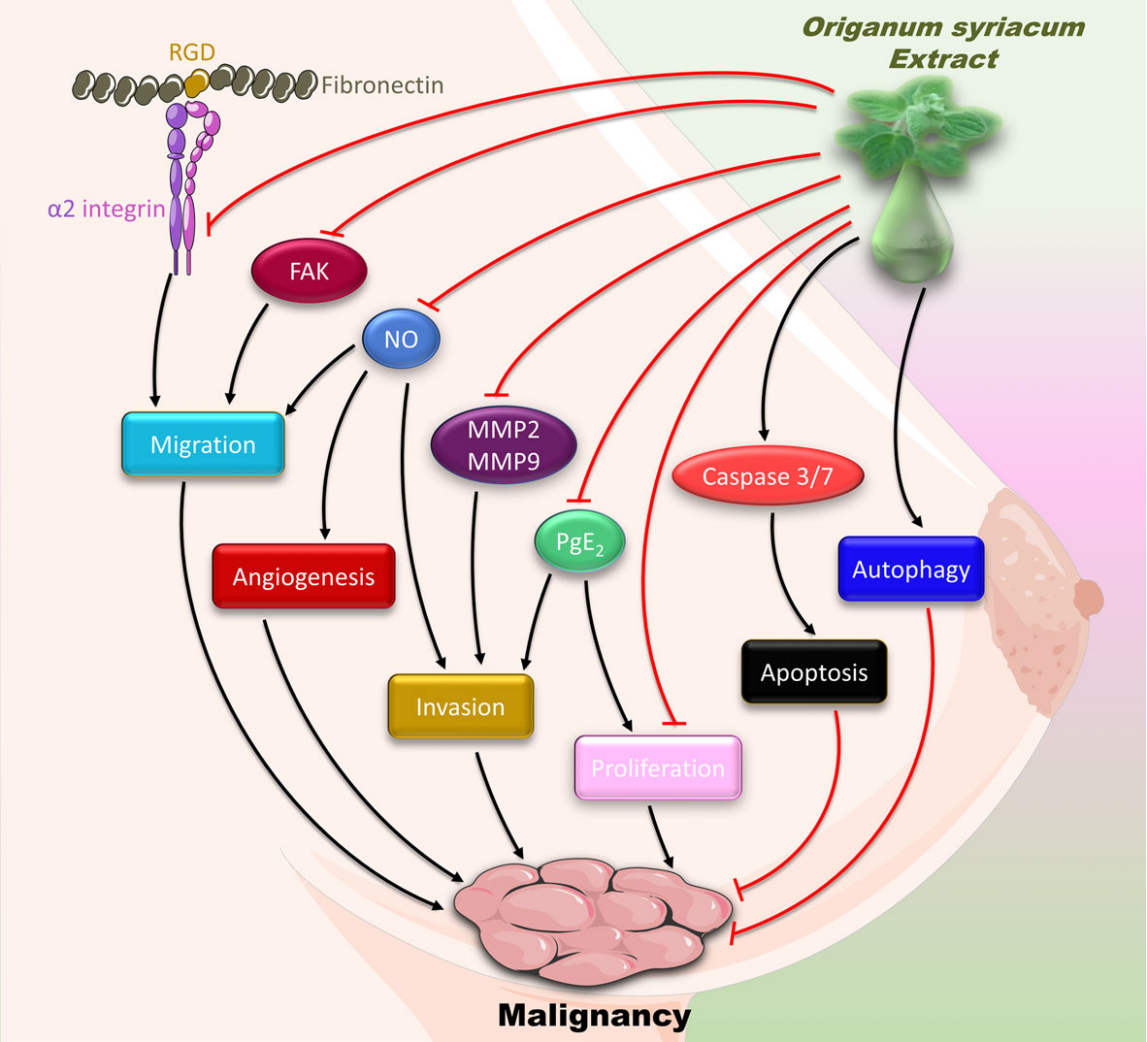
Summary of the anti-malignant mechanisms of OSE in MDA-MB-231 cells.

In conclusion, our results show that OSE inhibits the major hallmarks of cancer, namely proliferation, migration, invasion, and angiogenesis (**Figure 9**). These effects attenuate the malignant phenotype of MDA-MB-231cells, and thus may alleviate TNBC. However, further research is warranted to test OSE in an *in vivo* model and to dissect its molecular mechanism, especially that the protective effect of some natural compounds is controversial (72). Given the association between inflammation and cancer, assessing the anti-inflammatory effect of OSE would be interesting, especially that increased cytokine levels are linked to poor prognosis of TNBC. In addition, isolating and characterizing the bioactive molecule causing these OSE-induced anticancer and anti-inflammatory effects would be worth investigating. This is especially relevant since HPLC studies on *O. syriacum* have already been performed, and bioactives have been isolated long time ago (73). Some of these bioactives include apigenin, naringenin, rosmarinic acid, and caffeic acid (73). Interestingly, most of these bioactives possess potential anti-cancer effects. For instance, apigenin has been shown to suppress malignancy of MDAMB-231-derived tumors (74). Similarly, naringenin inhibits proliferation of MCF-7 and MDAMB-231 breast cancer cells (75). Rosmarinic acid inhibits proliferation and promotes apoptosis in MDAMB-231 and MDA-MB-468 cells, both being TNBC cells (76). Knowing that hormonal therapy is not sufficiently effective on TNBC and that it may impart resistance to chemotherapy, the search for alternative therapeutic approaches including herbal medicine becomes of utmost importance. It is critically important here to mention that oftentimes, the total extract or the whole herb provide more benefit than a single or a few bioactives. This may partly be due to inherent synergistic effects between the various bioactives, which could be lost when one or a few bioactives are separately consumed. Equally importantly, the plant is a typical part of the Mediterranean breakfast, and is consumed as a whole, not as individual ingredients per se. To the best of our knowledge, there is no clinical evidence to support a specific dose for consuming this plant. Nonetheless, this plant is well-known to be safe for human consumption (77), and is indeed one of the most consumed herbs in Lebanon and the Mediterranean region. Indeed, a study showed that the consumption of this plant’s essential oils had no adverse effects on animals (78) making it an attractive target for further studies that may eventually establish this herb as an important resource for potential drugs. However, the lack of *in vivo* animal studies is a limitation of this study, especially given the toxic effects of high doses of OSE in the developing embryo. Altogether, our study provides evidence for the *in vitro* anti-malignant effect of *O. syriacum* and opens new horizons to the potential of this plant as a source of bioactives that possess anti-cancer effects.

## Data Availability Statement

The raw data supporting the conclusions of this article will be made available by the authors, without undue reservation.

## Author Contributions

Conceptualization, AE; methodology, AA, MF, JM, RA, AB, EB, and SB; validation, FK, AE, SB, and EB; formal analysis, MF; investigation, AA, MF, JM, RA, and RI; resources, AB, EB, SN, and AE; data curation, AA, MF, JM, and RA.; writing— AA, MF, JM, RA, RI, SB, EB, AS, and SN; writing—review and editing, KM and AE; supervision, EB, AE, FK, RI, and AB; project administration, AE; funding acquisition, KM, AE, EB, AB, FK. All authors have read and agreed to the published version of the manuscript.

## Funding

This work was supported by funds from Qatar National Research Fund (UREP 16-145-3-030 to A.A and A.H.E), Qatar University fund (QUST-CAS-FALL-13/14-9 to A.A. and A.H. E.), the American University of Beirut (MPP #320133 (AE) and URB (EB)), University of Petra (AB, EB, and AE), and UAE University, UAE (funds#G3458 and G3908 to KM).

## Conflict of Interest

Author AA was employed by Kurome Therapeutics.

The remaining authors declare that the research was conducted in the absence of any commercial or financial relationships that could be construed as a potential conflict of interest.

## Publisher’s Note

All claims expressed in this article are solely those of the authors and do not necessarily represent those of their affiliated organizations, or those of the publisher, the editors and the reviewers. Any product that may be evaluated in this article, or claim that may be made by its manufacturer, is not guaranteed or endorsed by the publisher.
